# TAK1 expression is associated with increased PD-L1 and decreased cancer-specific survival in microsatellite-stable colorectal cancer

**DOI:** 10.1016/j.tranon.2024.102064

**Published:** 2024-07-27

**Authors:** Norman J. Galbraith, Jean A. Quinn, Sara Sf Al-Badran, Kathryn A.F. Pennel, Lily V.S. Hillson, Phimmada Hatthakarnkul, Molly McKenzie, Noori Maka, Lynette Loi, Mikaela Frixou, Colin W. Steele, Campbell S. Roxburgh, Paul G. Horgan, Donald C. McMillan, Joanne Edwards

**Affiliations:** a School of Cancer Sciences, Wolfson-Wohl Cancer Research Centre, University of Glasgow, Glasgow, United Kingdom; b Academic Unit of Surgery, School of Medicine, University of Glasgow, Glasgow Royal Infirmary, Glasgow, United Kingdom; c Department of Pathology, Queen Elizabeth University Hospital, Glasgow, United Kingdom

**Keywords:** Colorectal cancer, NFkappaB, Inflammation, PD-L1, Recurrence

## Abstract

**Background:**

Transforming growth factor β-activated protein kinase-1 (TAK1) plays an important role in MAPK and NFκB pathways and has been associated with colorectal cancer. The aim of this study was to determine how cytoplasmic and juxtanuclear punctate staining of TAK1 relates to immune checkpoint expression and cancer specific survival in colorectal cancer.

**Methods:**

Protein expression was assessed by immunohistochemistry on tissue microarrays from primary curative colorectal cancer resected specimens. Expression levels of cytoplasmic TAK1 by QuPath digital quantification and punctate TAK1 staining was scored using a manual point scoring technique and correlated with clinicopathological features, immune checkpoint expression and cancer-specific survival. Bulk RNA sequencing was performed in specimens to determine mutational profiles and differentially expressed genes.

**Results:**

A cohort of 875 patients who had undergone colorectal cancer resection were assessed for TAK1 expression. Higher levels of cytoplasmic TAK1 expression correlated with elevated PD1 and PD-L1 expression (*p* < 0.010). High punctate TAK1 expression was more commonly identified in poorly differentiated colorectal cancers (*p* = 0.036), had dysregulated mutational and transcriptional profiles with decreased insulin-like growth factor 2(*IGF2*) expression (*p* < 0.010), and independently predicted poor cancer-specific survival (HR 2.690, 95% CI 1.419–5.100, *p* = 0.002). The association of punctate TAK1 expression and recurrence remained after subgroup analysis for microsatellite-stable colorectal cancer (*p* = 0.028).

**Discussion:**

Punctate TAK1 expression is associated with worse cancer specific survival. TAK1 signalling may be an important pathway to investigate underlying mechanisms for recurrence in microsatellite-stable colorectal cancer.

## Highlights

• There is an unmet need for novel target discovery in microsatellite-stable colorectal cancer.
• A cohort of 875 patients with resected colorectal cancer specimens underwent immunohistochemistry for TAK1 expression.
• High levels of punctate TAK1 expression were associated with increased PD-1 and PD-L1 expression.
• Punctate TAK1 expression independently predicted poorer cancer-specific survival, including for subgroup analysis of microsatellite stable colorectal cancer.

## Introduction

Colorectal cancer remains the third most common cancer and 5-year survival for all patients remains around 60% [1,2]. While early-stage cancers and favourable biology permit a surgical cure, high-risk stage II, stage III and IV disease are associated with poor outcomes. The nuclear factor kappa-light-chain-enhancer of activated B cell (NFκB) pathway is considered a ubiquitous but critical pathway of inflammation in health and disease and has been associated with colorectal carcinogenesis [3]. Specifically, higher levels of NFκB subunits have been associated with tumour formation and worse survival [[4], [5], [6]].

Inhibition of the NFκB pathway has demonstrated sensitisation to 5FU, and specifically TAK1 inhibition can unlock chemoresistance to oxaliplatin [7,8]. However, the NFκB pathway is complex, redundant and with multiple levels of regulation, as well as crosstalk with other signalling pathways. As a result, characterising the dysregulation and aberrant specific pathways remains poorly understood and as a result, targeted therapy for NFκB has not yet been successfully translated into therapy. Targeting canonical or IκKβ has been poorly tolerated and unsuccessful due to its pleiotropic and fundamental role in cellular function, and more selective pathway manipulation is likely required. Our group has previously demonstrated the prognostic significance of phosphorylation of TAK1 (transforming growth factor β-activated protein kinase-1), along with IκK alpha and beta activation in colorectal cancer [4].

TAK1 belongs to a family of mitogen-activated protein three kinases (MAP3Ks) and has important functions in MAPK signalling, IκK beta activation, and modulation of other pathways such as SMAD and STAT signalling, all which are important in colorectal cancer. Within the NFκB pathway, TAK1 is considered part of the canonical pathway in IL1-beta and TLR4 receptor related pathways through TRAF6, following phosphorylation, IκK activation and downstream translocation of canonical subunits to the nucleus for transcription of cytokines, pro-angiogenic, apoptotic and other pro-carcinogenic functions [9].

A recent study demonstrated that TAK1 expression plays a role in radiotherapy resistance by dictating AP-1 activation to produce prion proteins causing radioresistance. In this study, TAK1 inhibition increased radiosensitivity in neuroblastoma, colon, and breast tumour cell lines [10]. There has been recent interest in the clinical implications of punctate staining of IκK alpha (IκKα) staining [11,12]. It is hypothesised that spatial localisation of IκKα subunits within the Golgi apparatus reflects increased pathway activation and for this reason is associated with worse survival. Despite this, the association between punctate expression and cytoplasmic expression for NFκB members is unclear, and no association was observed in prior study. Interestingly, IκKα is traditionally more associated with non-canonical pathways, but evidence is emerging in its clinical relevance in the canonical pathway [13].

In this report, we aimed to determine if there was evidence of punctate staining of TAK1 in colorectal cancer, and if punctate staining of this canonical associated kinase would relate to survival in patients with primary colorectal cancer.

## Methods

### Cohort characteristics

Patients undergoing colorectal resection ranging from stage I to IV primary colorectal cancer within NHS Greater Glasgow & Clyde between 1997 and 2007 (*n* = 1030) were included in the study. Exclusion criteria included palliative resection, inflammatory bowel disease-related malignancy, neoadjuvant chemotherapy, and those which did not survive beyond 30 days following surgery. Clinicopathological data were retrospectively collected from electronic medical records, and maintained on a prospective database. Staging of colorectal cancer was performed according to the AJCC TNM system. All patients were discussed in a multidisciplinary team (MDT) meeting and stage II CRC deemed to be high risk, as well as stage III disease generally received adjuvant chemotherapy (see Table 1). The REMARK guidelines (Reporting recommendations for tumour marker prognostic studies) were followed [14]. Local ethical approval was confirmed via the West of Scotland Research Ethics Committee. Cancer-specific survival was defined as the time from the date of surgery until the date of death from colorectal cancer.

**Table 1:** Clinicopathologic characteristics of patients undergoing colorectal resection for colorectal cancer and the relationship between cytoplasmic TAK1 and punctate TAK1 expression.

		All *N* = 875 (%)	Low cytoplasmic TAK1 *n* = 431 (%)	High cytoplasmic TAK1 *n* = 436 (%)	*p*	Low punctate TAK1 *n* = 589 (%)	High punctate TAK1 *n* = 286 (%)	*p*
**Age**	**<65**	259 (30)	140 (32)	177 (41)		177 (30)	82 (29)	
	**>65**	616 (70)	291 (68)	319 (59)	0.069	412 (70)	204 (71)	0.675
**Sex**	**Female**	309 (41)	213 (49)	223 (51)		309 (52)	129 (45)	
	**Male**	438 (59)	218 (51)	213 (49)	0.611	280 (48)	157 (54)	**0.041**
**Type**	**Elective**	687 (79)	349 (81)	334 (77)		478 (81)	209 (73)	
	**Emergency**	187 (21)	81 (19)	102 (23)	0.100	110 (19)	77 (27)	**0.005**
**Location**	**Right**	383 (44)	201 (47)	181 (42)		270 (46)	113 (40)	
	**Left**	300 (34)	137 (32)	156 (36)		188 (32)	112 (40)	
	**Rectum**	187 (21)	91 (21)	97 (22)	0.295	127 (22)	60 (20)	0.094
**T stage**	**1/2**	142 (16)	75 (17)	66 (15)		105 (18)	37 (13)	
	**3**	472 (54)	229 (53)	266 (61)		318 (54)	154 (54)	
	**4**	261 (30)	127 (29)	133 (30)	0.828	166 (28)	95 (33)	0.203
**N**	**0**	544 (62)	266 (52)	273 (63)		372 (63)	172 (60)	
	**1**	226 (26)	111 (26)	112 (26)		144 (24)	82 (29)	
	**2**	102 (12)	51 (12)	51 (12)	0.794	70 (12)	31 (11)	0.516
**M**	**0**	852 (97)	415 (96)	429 (98)		577 (98)	272 (95)	
	**1**	19 (3)	12 (4)	7 (2)	0.228	10 (2)	9 (5)	0.165
**Differentiation**	**Mod/well**	780 (89)	387 (90)	384 (88)		516 (88)	264 (92)	
	**Poor**	95 (11)	44 (10)	52 (12)	0.420	73 (12)	22 (8)	**0.036**
**Venous invasion**	**No**	580 (66)	275 (63)	297 (68)		390 (66)	190 (66)	
	**Yes**	295 (34)	156 (37)	139 (32)	0.180	199 (34)	96 (34)	0.949
**Margin involvement**	**No**	827 (94)	404 (94)	414 (95)		554 (94)	272 (95)	
	**Yes**	48 (6)	27 (6)	22 (5)	0.437	35 (6)	13 (5)	0.395
**Necrosis**	**Low**	541 (62)	252 (58)	268 (66)		345 (59)	178 (62)	
	**High**	340 (38)	173 (42)	158 (34)	0.279	232 (41)	103 (38)	0.317
**Status**	**Alive**	319 (36)	159 (37)	149 (34)		220 (37)	90 (31)	
	**Non-cancer death**	274 (31)	134 (32)	150 (34)		193 (33)	94 (34)	
	**Cancer related death**	290 (33)	130 (31)	130 (32)	0.553	163 (28)	100 (35)	0.074

P-value bold if <0.050.

Routine clinical laboratory haematological and biochemical parameters were prospectively recorded from electronic clinical records, and the Modified Glasgow Prognostic Score (mGPS) was calculated as either having a CRP <10 mg/L (scored as 0), CRP >10 mg/L and albumin >35 g/L (scored as 1), or CRP >10 mg/L but albumin <35 g/L (scored as 2) in keeping with previous reports [15].

### Immunohistochemistry

**Fig. 1:**
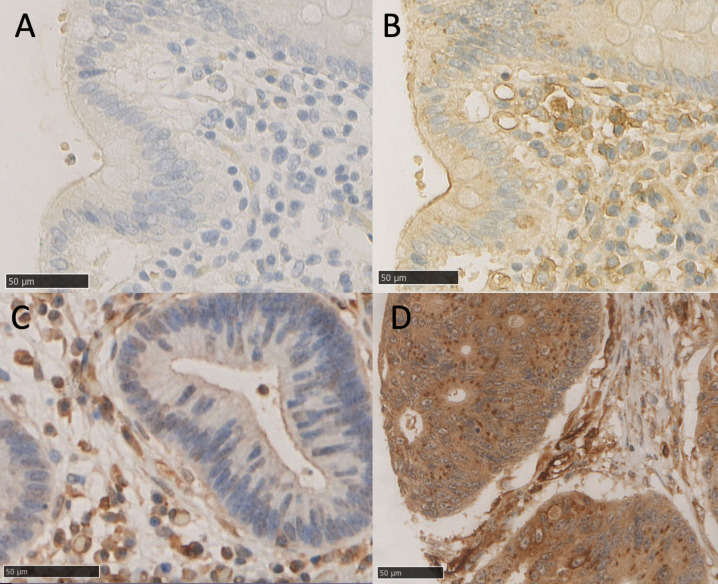
**TAK1 expression in colorectal cancer.** Representative chromogenic immunohistochemistry images of TAK1 staining showing a negative control (A), positive control (B), low cytoplasmic TAK1 expression (C), and high cytoplasmic TAK1 staining (D). Scale bars show 5 mm for full sections (A, B) and 50 µm for TMA images (C, D).

Formalin-fixed paraffin-embedded blocks were retrieved from archival tissue from the NHS Scotland Biorepository with three 0.6 mm cores selected from different areas of each patient tumour sample to account for tumour heterogeneity. Tissue microarrays (TMA) were then constructed to assess the expression of proteins of interest. Antibody specificity was previously confirmed using western blotting to identify a single band of the predicted molecular weight [4]. TMA sections (2.5 µm) were dewaxed by immersion in Histoclear® then rehydrated through a series of alcohols. Heat induced antigen retrieval was performed in citrate buffer pH6 after which the sections were incubated in 3% hydrogen peroxide. Non-specific binding was blocked by incubation in 5% normal horse serum TAK1 primary antibody (Abcam ab111096) was prepared at a concentration of 1:250 in antibody diluent (Agilent, London, UK) and sections incubated with it overnight at 4º C. Antibodies conditions for PD1 (HPA035981, Atlas Antibodies, Bromma, Sweden) were at 1:100 concentration, and for PD-L1 (HPA-13,967, Sigma-Aldrich, Gillingham, UK) at 1:1250 concentration as previously reported[16]. Staining was visualized using EnVisionTM (Dako, Agilent) and 3,3′-diaminobenzidine (DAB, Vector Labs, Newark, CA, USA). Tissue was counterstained using Harris Haematoxylin before being dehydrated and mounted using DPX. Negative controls were used with all staining, and positive controls were used to confirm antibody effectiveness (Fig. 1).

### Digital pathology assessment

Assessment of protein expression was carried out on QuPath [17] as previously described in Al-Badran and Grant et al. [16]. In brief, slides were de-arrayed and stain vectors were estimated during pre-processing to increase the staining quality. This was followed by a cell detection step using watershed cell detection. Different tissue was annotated into different tissue types (mainly tumour and stroma). A random trees classifier was trained using a variety of features, and three intensity thresholds were set to represent negative, weak, moderate, and strong staining, providing scores between 0 and 300. Based on the features and intensity thresholds, a classifier was built, and applied to all slides (Supplementary Figure 8). To ensure accuracy and reproducibility, a blinded examiner performed visual assessments using the histoscore method which correlated with the QuPath analysis. The distribution of cytoplasmic TAK1 expression was normal, and the mean level of expression was used to categorise patients as high or low cytoplasmic TAK1. A histogram illustrates the distribution of expression (Supplementary Figure 8). PD1 and PD-L1 were scored with a similar fashion[16].

### Punctate pattern assessment

**Fig. 2:**
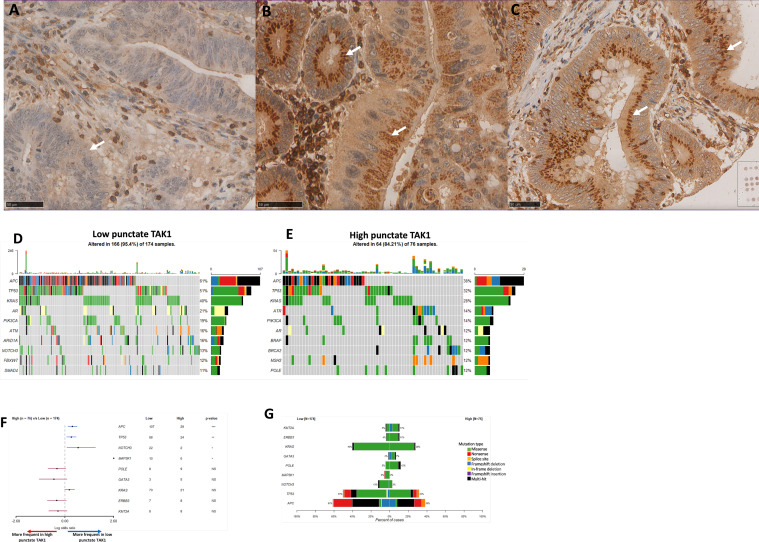
**Punctate TAK1 staining and mutational analysis in colorectal cancer.** Representative chromogenic immunohistochemistry images of TAK1 staining in tumour epithelium showing a absent/low punctate TAK1 staining (A), and then high punctate TAK1 expression (B, C). Scale bars show 50 µm for TMA images. Mutational analysis between high and low expression of cytoplasmic and punctate TAK1. Oncoplot of top 10 differentially mutated genes in low (D) and high (E) punctate TAK1 expression. (F) Forest plot comparing the top 10 differentially expressed genes between high and low punctate TAK1 expression. (G) Mutational type compared between high and low punctate TAK1 expression. * *p* < 0.050, **p < 0.01, *** *p* < 0.001. Note mutational analysis comparing high and low cytoplasmic TAK1 expression in Supplementary Data.

Punctate dots positive with TAK1 staining found within the tissue were assessed based on their size, number, and staining intensity, as per previous studies [11]. Based on criteria, cores were scored on a range of 0–3, where 0 is no punctate, 1 is weak punctate staining, 2 is moderate punctate staining, and 3 is strong punctate staining. TAK1 punctate staining was then categorised as high or low levels of staining via blinded microscopic examination using the Hamamatsu NDP Nanozoomer digital viewer program following electronic scanning of stained slides (Fig. 2). Missing cores and those containing less than 10% of tumour were excluded from analysis.

### Mutational analysis

Targeted capture sequencing was performed using RNA baits (Agilent Technologies, Santa Clara, CA, USA) to run an in-house custom panel of 151 cancer- related genes (Supplementary Material B). DNA was extracted from formalin-fixed paraffin-embedded sections from 237 stage I–IV CRC patients and standardised to a concentration of 4 ng/μl using the Qubit Assay (Thermo Fisher Scientific, UK). Targeted capture libraries were prepared from 150 to 200 ng DNA for sequencing using an Illumina HiSeq 4000 (Illumina, San Diego, CA, USA). This was performed by Glasgow Precision Oncology Laboratory. Gene mutation data were analysed and visualised using RStudio package ‘maftools’ (RStudio, Boston, <*A*, USA).

### RNA sequencing

Full transcriptomic RNA sequencing was performed using the Templated Oligo-Sequencing (Temp-O-Seq) platform from using a Whole Transcriptome panel (BioSpyder Technologies, Carlsbad, CA, USA). Formalin-fixed paraffin embedded sections were deparaffinised before digestion of tissue. Detector oligos were combined with tissue lysate for annealing together on the targeted RNA template and ligated. Amplification of the ligated oligos was performed with a unique primer set per sample by creating a unique barcode and adapters (Illumina, San Diego, USA). Coded samples were pooled into a single library and sequenced using the Illumina HiSeq 2500 High Output v4 flowcell. Reads were demultiplex using the BCL2FASTQ software (Illumina, San Diego, USA). FASTQ files were then aligned to the Human Whole Transcriptome v2.0 panel. This consisted of 22,537 probes, permitting up to two mismatches within the 50-nucleotide read. Methods are as described previously [18,19].

### Transcriptomic analysis

All transcriptomic analyses were performed using RStudio version 4.1.2. The Temp-O-Seq gene expression matrix was pre-processed to remove excess probes where there was more than one probe for a single gene by selecting the probe with the greatest mean expression across all samples. The package ‘DESeq2’ was used to find differential gene expression between TAK1 punctate ‘High’ and ‘Low’ samples [20]. Differential gene expression was visualised using ‘ggplot2’ and used to perform pairwise geneset enrichment analysis for the MSigDB Hallmark signatures using the ‘fgsea’ package[[21], [22], [23]]. Signatures with an adjusted p-value of <0.25 were plotted using ‘ggplot2’. The Microenvironment Cell Population Counter (MCPcounter) was performed on a vst normalised expression matrix. Single sample geneset enrichment analysis (ssGSEA) was performed on the vst normalised expression matrix using MSigDB Hallmark signatures in the ‘GSVA’ package[24]. Output from the MCPcounter and ssGSEA was visualised using ComplexHeatmap and ggplot2. A two-sided unpaired *t*-test assessed statistical significance in MCPcounter scores between groups.

### Statistical analysis

Chi Squared tests or Fisher's exact tests, where appropriate, were utilised to establish association between TAK1 protein expression and clinicopathological features. Kaplan-Meier curves and the log-rank test were used to assess the relationship between cytoplasmic and punctate TAK1 expression with 5-year cancer-specific survival. Univariate and multivariate associations between clinicopathologic parameters and cancer-specific survival was analysed using Cox-proportional hazards regression analysis to determine hazard ratio's (HR) and 95% confidence intervals (95% CI). Univariate variables that were found to have *p* < 0.05 were then entered to a multivariate Cox regression model. A p-value of <0.05 was used to demonstrate statistical significance. SPSS was used for statistical analysis (IBM SPSS, version 28, IBM Corp., Armonk, N.Y., USA). R studio was used for data visualisation (RStudio version 4.1.2, Boston, MA, USA). For mutational analysis, Fisher's exact test was used to compare the 10 most statistically significant genes in patients with low and high expression of cytoplasmic and punctate TAK1.

## Results

There were 1031 patients who underwent resection for colorectal cancer that were initially identified. There were 3.7% of patients who had undergone neoadjuvant chemotherapy and/or radiotherapy and thus excluded from analysis (Supplementary Figure 1). Patients with missing follow up data or inadequate tissue for histological assessment were also excluded. This permitted 875 patients eligible for analysis. Of these, 50.1% were male and 32.9% of the patients underwent adjuvant chemotherapy. Colon cancer was the location of the tumour in 74.6% of the patients, with 31.4% having rectal cancer. In over 70% of cases, patients were older than 65 years old at the time of resection, and in 21.3% of cases the resection was performed in the emergency setting.

There were 431 patients in each of the low and high cytoplasmic TAK1 groups. Most patients had low punctate TAK1 staining (*n* = 589, 67.3%), compared with high punctate TAK1 staining (*n* = 286, 32.7%). Most patients with either low or high cytoplasmic TAK1 expression had low punctate staining, however, there were more patients with high TAK1 punctate expression in the high cytoplasmic TAK1 group (*p* < 0.01) (Fig. 1& 2). Table 1 summarises the clinical demographics in patients with both high and low cytoplasmic TAK1 expression, and in patients with high and low punctate TAK1 staining. TAK1 punctate staining appeared to be higher in emergency cases compared with elective cases (*p* = 0.005), left sided cancers when compared with right or rectal cancers (*p* = 0.045), and in poorly differentiated cancers (*p* = 0.036). MMR deficiency was identified in 17.5% of patients (Table 2).

**Table 2:** Relationship between cytoplasmic and punctate TAK1 expression with tumour microenvironment immune parameters of colorectal cancer.

		All *N* = 875 (%)	Low cytoplasmic TAK1 *n* = 431 (%)	High cytoplasmic TAK1 *n* = 436 (%)	*p*	Low punctate TAK1 *n* = 589 (%)	High punctate TAK1 *n* = 286 (%)	*p*
**Microsatellite status**	**MSS**	533 (88)	265 (87)	262 (89)		342 (64)	191 (95)	
	**MSI**	74 (12)	41 (13)	32 (11)	0.383	191 (36)	10 (5)	**<0.001**
**Klintrup-Makinen grade**	**Weak**	586 (67)	284 (67)	296 (68)		396 (67)	190 (68)	
	**Strong**	274 (33)	142 (33)	131 (32)	0.406	183 (33)	91 (32)	0.818
**TSP**	**Low**	656 (77)	325 (76)	324 (76)		442 (77)	214 (77)	
	**High**	199 (23)	98 (24)	199 (24)	0.886	135 (23)	64 (23)	0.903
**Tumour budding**	**Absent**	574 (66)	277 (73)	291 (69)		386 (73)	188 (67)	
	**Present**	233 (34)	100 (27)	130 (31)	0.175	146 (27)	87 (33)	0.213
**TIL**	**Absent**	663 (78)	335 (79)	321 (75)		436 (76)	227 (81)	
	**Present**	192 (22)	86 (21)	106 (25)	0.126	138 (24)	54 (19)	0.112
**CD3 (stroma)**	**Low**	408 (49)	205 (51)	203 (48)		279 (50)	129 (47)	
	**High**	424 (51)	202 (49)	222 (52)	0.453	281 (50)	146 (53)	0.429
**CD8 (stroma)**	**Low**	500 (60)	245 (60)	254 (61)		335 (61)	165 (60)	
	**High**	330 (40)	158 (40)	169 (39)	0.826	219 (39)	111 (40)	0.849
**FOXP3**	**Low**	255 (35)	130 (37)	124 (32)		170 (35)	85 (34)	
	**Medium**	270 (37)	125 (36)	145 (37)		174 (36)	96 (39)	
	**High**	212 (28)	99 (28)	113 (29)	0.476	146 (29)	66 (27)	0.592
**CD66b (stroma)**	**Low**	109 (28)	52 (28)	56 (29)		67 (42)	42 (33)	
	**High**	274 (72)	135 (72)	136 (71)	0.769	188 (58)	86 (67)	0.181
**CD68 (stroma)**	**Low**	294 (56)	134 (57)	161 (55)		208 (58)	86 (53)	
	**High**	231 (44)	100 (43)	130 (45)	0.656	154 (42)	77 (47)	0.316
**CD45 (stroma)**	**Low**	158 (42)	58 (39)	95 (44)		103 (42)	50 (42)	
	**High**	219 (58)	94 (61)	117 (56)	0.205	142 (58)	68 (58)	0.952
**IκKα (cytoplasmic)**	**Low**	241 (33)	175 (49)	66 (17)		174 (36)	67 (27)	
	**Medium**	243 (34)	120 (35)	123 (32)		145 (31)	98 (39)	
	**High**	241 (33)	55 (16)	187 (51)	<0.001*	160 (33)	81 (32)	0.014*
**IκKα (nuclear)**	**Low**	254 (35)	106 (30)	149 (40)		172 (37)	82 (33)	
	**Medium**	235 (32)	111 (32)	124 (33)		153 (32)	82 (33)	
	**High**	236 (33)	133 (38)	103 (23)	0.004*	154 (33)	82 (33)	0.789
**IκKα (punctate)**	**Low**	158 (22)	87 (25)	71 (19)		150 (32)	8 (35)	
	**Medium**	243 (34)	104 (31)	139 (38)		208 (44)	35 (14)	
	**High**	320 (44)	156 (44)	165 (43)	0.054	118 (25)	202 (82)	<0.001*
**STAT1 (cytoplasmic)**	**Low**	131 (87)	36 (86)	88 (82)		82 (93)	42 (84)	
	**High**	14 (13)	6 (14)	8 (18)	0.287	6 (7)	8 (16)	0.086
**RelB (nuclear)**	**Low**	313 (44)	131 (39)	180 (49)		196 (43)	117 (46)	
	**High**	404 (56)	207 (61)	194 (51)	**0.012**	266 (57)	138 (54)	0.371
**RelB (cytoplasmic)**	**Low**	231 (32)	130 (38)	96 (25)		155 (33)	76 (22)	
	**High**	484 (68)	208 (62)	276 (75)	<0.001*	306 (67)	178 (78)	0.311
**HIF alpha**	**Low**	583 (66)	316 (73)	259 (60)		392 (66)	191 (67)	
	**High**	292 (34)	115 (27)	177 (40)	<0.001*	197 (34)	95 (33)	0.946
**PD1**	**Low**	99 (20)	59 (26)	38 (15)		67 (21)	32 (12)	
	**High**	384 (80)	163 (74)	217 (85)	**0.002**	252 (79)	132 (88)	0.701
**PD-L1**	**Low**	408 (46)	225 (52)	179 (41)		277 (47)	131 (46)	
	**High**	467 (54)	206 (48)	257 (59)	<0.001*	312 (53)	155 (54)	0.733

MSS; microsatellite stable, MSI; microsatellite instable,; TIL, tumour-infiltrating lymphocytes; TSP, tumour-stromal percentage. P-value bold if <0.050.

**Fig. 3:**
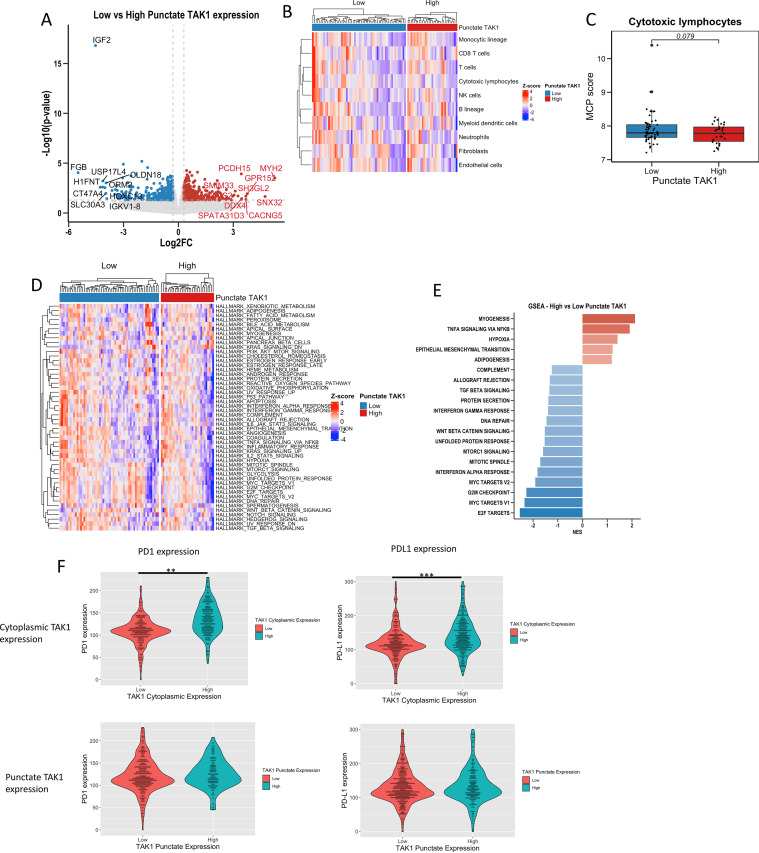
Transcriptional and immune checkpoint differences between low and high punctate TAK1 expression. (A) Volcano plot comparing differentially expressed genes. (B) Heatmap of differences in microenvironment immune cell populations and (C) comparison of cytotoxic lymphocyte populations. (D) Heatmap comparing differential expression of hallmarks of cancer pathways. (E) Z-plot comparing gene set enrichment scores based on punctate TAK1 expression. (F) Differences in PD1 (left) & PD-L1 (right) based on cytoplasmic (top) and punctate (bottom) TAK1 expression.

No differences in TAK1 cytoplasmic or punctate expression were demonstrated between patients with wild-type compared with mutant-type status for *p53, PI3K, BRAF* and *KRAS* (Supplementary Table 1). Mutational profiling analysis revealed that patients with low punctate TAK1 staining had higher levels of mutations in *APC* (61% vs. 38%), *TP53* (51% vs. 32%), and *NOTCH3* genes (*p* < 0.050) (Fig. 3). Interestingly, patients with higher punctate TAK1 levels appeared to have more prevalent mutations in *POLE, GATA* and *ERBB3*, however, these did not reach statistical significance.

RNA sequencing analysis was performed to investigate transcriptional differences between patients with high and low punctate TAK1 levels. On comparing differential expression of genes there was significantly downregulated insulin-like growth factor-2 (*IGF2*; padj < 0.0001, log2(fold-change) = −4.5) levels in high compared with low punctate TAK1 subgroups, even when adjusting for multiple hypothesis testing (Fig. 3A, Supplementary Figure 7). Immune cell deconvolution appeared to show a trend for decreased cytotoxic lymphocytes within the tumour microenvironment in patients with high punctate TAK1 expression (Fig. 3B, C; cytotoxic lymphocytes padj = 0.079). Upregulated pathways include myogenesis (NES = 2.12, padj < 0.0001), TNFA signalling via NFKB (NES = 0.91, padj < 0.0001, and epithelial-mesenchymal transition (NES = 1.22, padj < 0.25, whereas E2F (NES = −2.55, padj < 0.00001) and MYC targets (NES = −2/34, padj < 0.0001) were downregulated (Fig. 3D, E). Transcriptional differences based on cytoplasmic TAK1 expression is demonstrated in Supplementary Figure 6.

Interestingly, both cytoplasmic and punctate TAK1 expression were not associated with systemic inflammatory status, whether this was measured by levels of neutrophils, platelets, albumin, CRP, or various ratios of these parameters (Supplementary Table 1). While cytoplasmic TAK1 expression was similar between microsatellite stability status, high punctate TAK1 staining was more common in microsatellite stable patients (*p* < 0.001). Patients who had died from colorectal cancer had a higher expression of punctate TAK1 staining, although this was not statistically significant (*p* = 0.074). Furthermore, there were no significant differences in the cellular constitution of the tumour microenvironment, with immune infiltrate as measured by the Klintrup-Makinen grade or by staining for CD3, CD8, CD66b, CD68, FOXP3 and tumour stromal percentage (Supplemental Figure 2). The lower presence of TIL's identified on IHC (23.7% compared with 31.6%) did not reach statistical significance (*p* = 0.112).

There were no obvious relationships between TAK1 and levels of immune infiltrate, tumour-stromal percentage or immune cell subsets (Table 2). Cytoplasmic TAK1 expression was associated with markers of inflammatory signalling biomarkers expressed within the tumour cells and cytoplasmic TAK1 expression was associated with higher cytoplasmic and nuclear IκKα expression (*p* < 0.005). Further NFκB -related components, RelB and HIF1 alpha (HIF-1α), were also associated with high cytoplasmic TAK1 expression (*p* < 0.001). Patients with high cytoplasmic TAK1 were also found to have higher levels of immune checkpoint proteins, PD1 and PD-L1 (*p* < 0.005) (Fig. 5). High punctate TAK1 expression was associated with IκK in the cytoplasmic and punctate forms (*p* = 0.014 and <0.001, respectively).

**Fig. 4:**
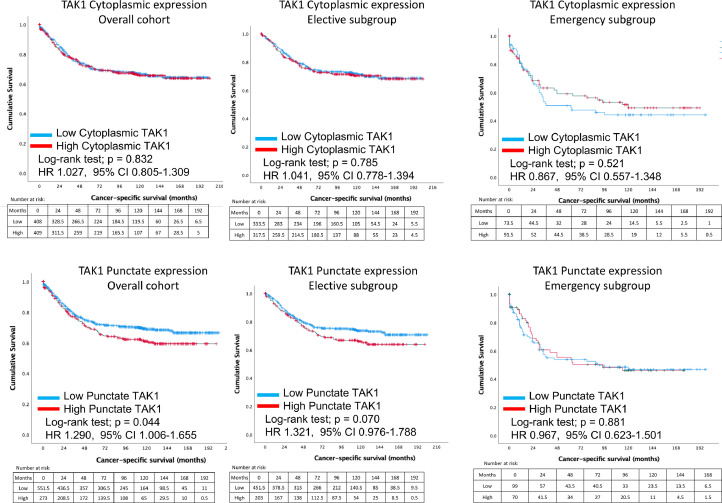
Differences on cancer specific survival between patients with high or low TAK1 cytoplasmic (top) and punctate (bottom) staining. Comparisons between low expressors (blue) and high expressors (red) are demonstrated on separate survival curves. The total cohort is show on the left, and then subgroup analysis of patients that underwent elective and emergency resection, respectively, are shown.

There were no differences in cancer-specific survival between high and low TAK1 cytoplasmic expression when assessed in the full cohort. Fig. 4 shows Kaplan-Meier curves and long-rank analysis demonstrating decreased cancer-specific survival in patients with higher TAK1 punctate expression (*p* = 0.044). Those patients with high punctate TAK1 expression had a mean survival of 134.8 months (95% 124.7–145.1 months) compared with a mean survival of 149.1 months (95% CI 142.0 – 156.1 months) in patients with low punctate TAK1 expression. Life table analysis demonstrated 5-year survival for those with high punctate TAK1 expression was 59.4%, compared with 71.5% in patients with low punctate TAK1 expression.

**Fig. 5:**
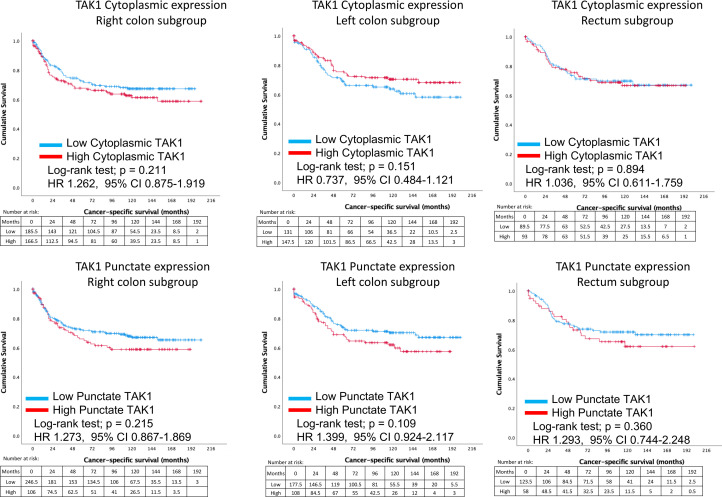
Differences in cancer-specific survival between patients with high and low TAK1 cytoplasmic (top) and punctate (bottom) expression, categorised based on location of colorectal primary tumour (right, left and rectum).

Subgroup analysis was performed to compare elective and emergency cases (Fig. 4). Regardless, of the acuity of presentation, TAK1 cytoplasmic staining did not significantly influence cancer-specific survival. On determining the influence of TAK1 punctate staining, subgroup analysis demonstrated that similar patterns emerged in elective resection with higher TAK1 punctate staining leading to poorer survival, although not reaching statistical significance within subgroups (*p* = 0.075). When comparing TAK1 cytoplasmic and punctate expression within subgroups of right colon, left colon and rectum, differences in cancer-specific survival did not reach statistical significance in these smaller groups. It is of note that for left sided cancers, higher cytoplasmic TAK1 expression seems to be protective, in contrast to right sided cancers (Fig. 5). A trend for high TAK1 punctate staining and worse cancer-specific survival regardless of tumour location.

**Fig. 6:**
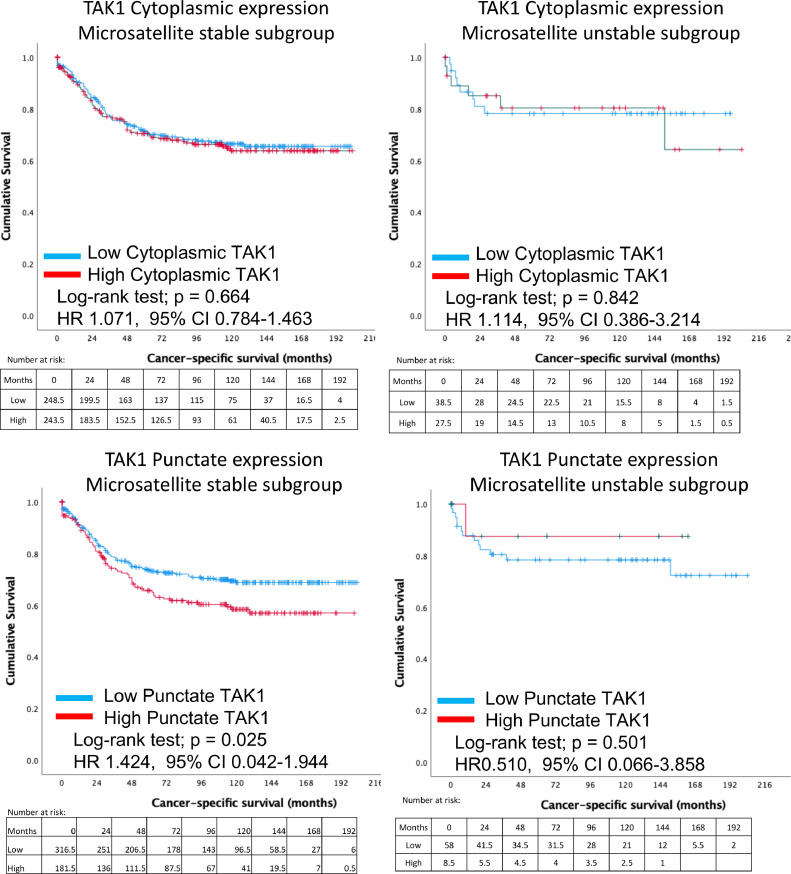
Comparisons between cancer-specific survival between high and low expressing TAK1 cytoplasmic (top) and punctate (bottom) patients are shown, based upon microsatellite stability status. MSS, microsatellite stable; MSI, microsatellite instable.

More importantly, when categorised into microsatellite stable patients, high TAK1 punctate expression was significantly associated with worse cancer-specific survival (*p* = 0.028) (Fig. 6). When patients specifically with MSS status only were analysed, patients with low punctate TAK1 expression had a mean survival of 149.9 months (95% CI 140.7 – 159.1 months) compared with only 130.7 months (95% CI 117.9 – 143.5 months) in patients with high punctate TAK1 expression. This equates to a 5-year survival of 68.8% in low punctate TAK1 expression compared with 57.0% in patients with a high punctate TAK1 status.

On univariate regression analysis, TNM staging, poor differentiation, positive margin involvement, peri‑tumoural immune infiltrate (as measured by Klintrup-Makinen), tumour stromal percentage and tumour budding were all predictive of cancer-specific mortality (*p* < 0.001). Table 3 demonstrates that punctate IκKα and TAK1 staining, as well as PD1 expression were also predictive of cancer-specific mortality. Multivariate regression analysis was performed on all significant predictors on univariate analysis. This analysis was demonstrated that nodal and metastatic status, positive margins, and high TAK1 punctate staining remained significant predictors of cancer-specific death in colorectal cancer (*p* < 0.050). It is of note that following multivariate analysis, PD1 had a hazard ratio of 0.606 (CI 0.361–1.016, *p* = 0.058) in relation to cancer-specific survival.

**Table 3:** Univariate and multivariate regression analysis of TAK1 expression and clinical and tumour microenvironment parameters on cancer-specific survival in patients undergoing surgery for colorectal cancer.

		Cancer-specific survival			
Variable		Univariate	*p*	Multivariate	*p*
Age	≤65 (ref)	1.0			
	>65	1.137 (0.893–1.448)	0.298		
Sex	Female (ref)	1.0			
	Male	1.127 (0.899–1.414)	0.299		
Adjuvant therapy	No (ref)	1.0			
	Yes	1.086 (0.725–1.627)	0.689		
Location	Colon	1.0			
	Rectum	0.903 (0.689–1.182)	0.457		
T-stage	1 (ref)	1.0			
	2	0.964 (0.377–2.463)	0.938		
	3	2.021 (0.893–4.573)	0.091		
	4	4.163 (1.837–9.436)	<0.001*	2.629 (0.338–20.412)	0.355
N-stage	0 (ref)	1.0			
	1	2.602 (0.021–3.351)	<0.001*		
	2	3.857 (2.835–5.248)	<0.001*	2.224 (1.201–4.120)	0.011*
M-stage	0 (ref)	1.0			
	1	7.362 (4.594–11.796)	<0.001*	3.380 (1.348–8.477)	0.009*
Differentiation	Well/moderate (ref)	1.0			
	Poor	1.902 (1.390–2.605)	<0.001*	1.142 (0.587–2.223)	0.695
Venous invasion	No (ref)	1.0			
	Yes	2.153 (1.717–2.700)	<0.001*	1.423 (0.887–2.281)	0.143
Margin Involv.	No (ref)	1.0			
	Yes	3.110 (2.173–4.450)	<0.001*	2.690 (1.256–5.761)	0.011*
mGPS	Low (ref)	1.0			
	High	3.419 (2.538–4.606)	<0.001*	3.114 (1.695–5.719)	<0.001
KM grade	Low (ref)	1.0			
	High	0.361 (0.266–0.491)	<0.001*	327.773 (0.000–3.866^44^)	0.907
TSP	Low (ref)	1.0			
	High	1.876 (1.473–2.388)	<0.001*	1.881 (0.558–6.341)	0.308
Tumour budding	Low (ref)	1.0			
	High	1.359 (1.025–1.743)	0.016*	1.393 (0.849–2.286)	0.190
MSS status	MSS (ref)	1.0			
	MSI-H	0.597 (0.353–1.011)	0.055		
TAK1 Cytoplas.	Low (ref)	1.0			
	High	1.027 (0.805–1.309)	0.832		
TAK1 Punctate	Low (ref)	1.0			
	High	1.290 (1.006–1.655)	0.045*	2.690 (1.419–5.100)	0.002*
IκK Cytoplasmic	Low (ref)	1.0			
	High	1.147 (0.866–1.519)	0.339		
IκK Nuclear	Low (ref)	1.0			
	Medium/High	0.963 (0.820–1.132)	0.651		
IκK Punctate	Low (ref)	1.0			
	Medium/High	0.607 (0.411–0.898)	0.012*	0.579 (0.272–1.236)	0.158
PD1	Low (ref)	1.0			
	High	0.608 (0.424–0.871)	0.007*	0.606 (0.361–1.016)	0.058
PD-L1	Low (ref)	1.0			
	High	0.872 (0.696–1.092)	0.234		

mGPS, modified Glasgow Prognostic Score; KM, Klintrup-Makinen; TNM, tumour, nodes, metastasis, respectively; MMR, mismatch repair status. TSP, tumour-stromal percentage. P-value bold if <0.050.

## Discussion

Colorectal cancer remains a major cause of death and most current therapies for advanced disease remains a “one size fits all” approach with only a small proportion of patients eligible for immunotherapy. It is critical to gain a better understanding of the changes in inflammatory signalling pathways that lead to poorer outcomes. This report explores the expression of cytoplasmic and punctate patterns of TAK1 expression in patients with colorectal cancer. Punctate TAK1 expression was found to be higher in emergency cases and tumours with poorer differentiation. Microsatellite-instability high tumours were more likely to lower levels of punctate TAK1 staining, but expression was similar between other mutational statuses. Systemic inflammatory status was not associated with TAK1 expression. Within the tumour epithelial cells, however, various NFκB related signalling pathways had corresponding upregulation such as IκKα, RelB and HIF1α. High cytoplasmic TAK1 expression was associated with higher PD1 and PD-L1 expression in tumours. Punctate TAK1 expression was predictive of cancer-specific mortality in colorectal cancer.

Both punctate forms of TAK1 within the tumour, alongside IκKα, correlate with not just each other, but also appear to independently predict death after colorectal cancer resection. Our understanding of the pathophysiological process for the accumulation of these proteins is incomplete. Increased punctate staining may be a surrogate marker for increased activation, and thus subcellular processing and recycling of degradation products, within this pathway. Patel et al. demonstrated that punctated forms of IκKα were observed in discrete juxtanuclear punctate areas which co-localised to the Golgi apparatus or a Golgi-related structure [11]. An alternative explanation would be that increased TAK1 punctate expression plays a role in the over-activation or dysfunction of a tumoural cell and is causal in promoting cellular malfunction such as defective autophagy. In the present study, punctate and cytoplasmic forms do correlate for TAK1 which in is in contrast to Patel et al., where a relationship between cytoplasmic and punctate IκKα was not observed.

In patients with high punctate TAK1 staining, given their worse outcome, the lower frequency of *APC* and *TP53* mutations when compared to low punctate staining was unexpected. One might speculate that by comparison to a more traditional mutational profile seen in the low punctate group, the smaller group of high punctate TAK1 patients have an alternative profile of unconventional mutations, such as *POLE*, which may be a cause of increased activation of these inflammatory pathways which is seen by increased punctate staining and in turn less favourable outcomes. Indeed, the mutational profiles for colorectal cancer vary on location of the tumour, and even when controlling for microsatellite stable tumours, punctate TAK1 predictors worse cancer-specific mortality. Interestingly, cytoplasmic TAK1 does have some influence on survival, however, the effect appears to be positive or negative depending on microsatellite status and location throughout the colon (Supplementary Figure 4).

TAK1 is an important protein within the MAPK pathway but has critical roles in the activation of the TGF-beta, NFκB and MAPK pathway, as well as functioning upstream of various SMAD and STAT proteins [25]. As a result, TAK1 can be activated by stress signals, cytokines, hypoxia and DNA damage. MUC1 which is associated with high mucin-related colorectal cancer has been shown to activate TAK1 expression in colorectal cancer, driving NFκB towards inflammation-associated cancer progression [26]. miRNA-215 appears to be one epigenetic mechanism governing TAK1, with reciprocal expression in colorectal cancer [27]. While the effect is context specific, it is unsurprising that dysregulation of TAK1 can lead to cell cycle and differentiation, immune response, and homoeostasis. Depending on said context, TAK1 can function as pro-tumour or anti-tumour. This understanding certainly explains the crosstalk with other signalling pathways in tumours within this dataset. TAK1-specific therapeutic targeting has been investigated with the development of Takinib which in some early data can induce tumour remission[28] and abrogate chemoresistance [29]. Further recent data has shown that TAK1 targeting can contribute to chemoresistance in colorectal cancer due to cancer-associated fibroblasts [7]. The primary effect of TAK1 inhibition appears to relate to a promoting apoptosis of cancer cells and prevention of tumour growth [[30], [31], [32], [33]]. TAK1 inhibition in tumour cells has been shown to increase heat shock proteins, caspases, and associated with necroptosis. Our data demonstrates that high levels of TAK1 expression in the punctate form is associated with upregulated downstream NFκB signalling via IκKα and HIF1 alpha which is associated with a poorer cancer-related outcome. Interestingly, the association of RelB with TAK1 expression in our data may suggest that the alternative NFκB pathway is complicit in colorectal cancer pathogenesis, which is in keeping with the literature [34]. Importantly, our data demonstrates that for patients who undergo resection and then adjuvant chemotherapy, longer term survival appears better in patients where the levels of cytoplasmic and punctate TAK1 are high in the specimen (Supplementary Figure 3).

RNA sequencing analysis in our data demonstrated that patients with high punctate TAK1 levels have lower levels of *IGF2. IGF2* has been implicated in the development of colorectal cancer through an epigenetic alteration from loss of imprinting [35]. *IGF2* has been used as part of a panel to test for CpG island methylator phenotype (CIMP) colorectal cancers which were associated with worse survival [36,37]. Experimental studies have shown that colon cancer cell lines co-cultured with cancer-associated fibroblasts overexpressing *IGF2* demonstrated increased invasiveness [38]. A recent report has demonstrated that differences in *IGF2* methylation in peripheral blood leukocytes can confer increased risk of colorectal cancer [39]. Given the significant role of epigenetics in the regulation of *IGF2*, then further studies are warranted to understand the differences in *IGF2* gene expression and downstream protein and functional effects of these differences.

In conclusion, we have demonstrated that TAK1 expression in colorectal cancer is associated with NFκB pathway and immune checkpoint upregulation. High punctate TAK1 expression occurs in poorly differentiated tumours and independently predicts mortality after surgical resection for colorectal cancer. This data provides the clinical rationale for therapeutic targeting of TAK1 at this level, or within the NFκB pathway. Given the higher proportion of patients with high punctate TAK1 expression in microsatellite-stable patients who currently would not be eligible for immunotherapy, high punctate TAK1 may represent a marker to guide targeted therapies in this subset of patients.

## Ethics approval

Ethical approval for use of patient tissue samples was gained from the NHS Greater Glasgow & Clyde Tissue Biorepository (biobank ethical approval number is 10/50,704/ 60, and the safe haven ethical approval number is 12/ws/0142). Patient database is held by the NHS Greater Glasgow & Clyde Safe Haven under GSH/18/ON/007. The study was performed in accordance with the Declaration of Helsinki. Informed consent was obtained from all patients.

## Consent for publication

Not applicable - No individualised patient data has been used.

## Data availability

All data generated in this study are available on reasonable request to the senior author.

## Fundings information

NG is funded by the Chief Scientists Office (Scotland) (PCL/21/03). KAFP is funded by Chief Scientists Office (Scotland) (TCS/22/02). CSW is funded by UKRI (MR/WOO7851/1). CSR is funded by Chief Scientists Office (Scotland) (SAF/19/01). JE is supported by CRUK (CTRQQR-2021/1000006).

## CRediT authorship contribution statement

**Norman J. Galbraith:** Writing – review & editing, Writing – original draft, Visualization, Validation, Supervision, Project administration, Methodology, Investigation, Funding acquisition, Formal analysis, Data curation, Conceptualization. **Jean A. Quinn:** Writing – review & editing, Methodology, Data curation, Conceptualization. **Sara Sf Al-Badran:** Writing – review & editing, Project administration, Formal analysis. **Kathryn A.F. Pennel:** Writing – review & editing, Methodology, Investigation. **Lily V.S. Hillson:** Writing – review & editing, Visualization, Methodology. **Phimmada Hatthakarnkul:** Writing – review & editing, Visualization, Methodology. **Molly McKenzie:** Writing – review & editing. **Noori Maka:** Validation, Methodology. **Lynette Loi:** Methodology. **Mikaela Frixou:** Methodology. **Colin W. Steele:** Writing – review & editing. **Campbell S. Roxburgh:** Writing – review & editing. **Paul G. Horgan:** Writing – review & editing, Funding acquisition, Conceptualization. **Donald C. McMillan:** Writing – review & editing. **Joanne Edwards:** Writing – review & editing, Supervision, Resources, Project administration, Funding acquisition, Conceptualization.

## Declaration of competing interest

The authors declare that they have no known competing financial interests or personal relationships that could have appeared to influence the work reported in this paper.

## Appendix Supplementary materials

Image, application 1

Image, application 2
