# Supplementary material for: TAK1 expression is associated with increased PD-L1 and decreased cancer-specific survival in microsatellite-stable colorectal cancer
Source: Transl Oncol. 2024 Jul 27;48:102064. doi: 10.1016/j.tranon.2024.102064 (PMC11338118; doi:10.1016/j.tranon.2024.102064)
Supplement: Supplementary file 2 [file mmc2.docx]

**Supplementary Table 1.** Relationship between cytoplasmic and punctate TAK1 expression with mutational status and systemic inflammatory status.

*mGPS, modified Glasgow Prognostic Score; TNM, tumour, nodes, metastasis, respectively; MMR, mismatch repair status. P-value bold if <0.050.*

|  |  | **All**  **N=906** | **Low cytoplasmic TAK1**  **n=449** | **High cytoplasmic TAK1**  **n=449** | ***p*** | **Low punctate TAK1**  **n=615** | **High punctate TAK1**  **n=291** | ***p*** |
| --- | --- | --- | --- | --- | --- | --- | --- | --- |
| **BRAF** | **WT** | 198 | 100 | 98 |  | 124 | 76 |  |
|  | **Mutant** | 31 | 18 | 13 | 0.434 | 23 | 8 | 0.189 |
| **KRAS** | **WT** | 114 | 62 | 52 |  | 78 | 37 |  |
|  | **Mutant** | 115 | 56 | 59 | 0.389 | 69 | 47 | 0.187 |
| **PI3K** | **WT** | 31 | 12 | 19 |  | 19 | 13 |  |
|  | **Mutant** | 12 | 3 | 9 | 0.398 | 7 | 5 | 0.950 |
| **P53** | **WT** | 20 | 5 | 15 |  | 14 | 7 |  |
|  | **Mutant** | 23 | 10 | 13 | 0.205 | 12 | 11 | 0.329 |
| **mGPS** | **0** | 385 | 186 | 194 |  | 260 | 125 |  |
|  | **1** | 206 | 100 | 105 |  | 132 | 74 |  |
|  | **2** | 137 | 77 | 58 | 0.233 | 97 | 30 | 0.420 |
| **CRP** | **Normal** | 377 | 184 | 193 |  | 258 | 124 |  |
|  | **High** | 339 | 177 | 162 | 0.353 | 228 | 114 | 0.803 |
| **Albumin** | **Normal** | 593 | 295 | 298 |  | 398 | 201 |  |
|  | **Low** | 177 | 101 | 76 | 0.087 | 124 | 55 | 0.480 |
| **Platelets** | **Normal** | 525 | 288 | 237 |  | 359 | 173 |  |
|  | **High** | 114 | 61 | 53 | 0.793 | 72 | 43 | 0.315 |
| **Neutrophils** | **Normal** | 584 | 309 | 275 |  | 398 | 192 |  |
|  | **High** | 145 | 77 | 68 | 0.967 | 96 | 51 | 0.620 |
| **Lymphocytes** | **Normal** | 727 | 385 | 342 |  | 493 | 242 |  |
|  | **Low** | 2 | 1 | 1 | 0.933 | 1 | 1 | 0.608 |
|  |  |  |  |  |  |  |  |  |
| **Neutrophil-lymphocyte ration (NLR)** | **Low** | 523 | 285 | 238 |  | 353 | 173 |  |
|  | **High** | 205 | 100 | 105 | 0.165 | 140 | 70 | 0.908 |
| **Neutrophil-platelet score (NPS)** | **Low** | 434 | 238 | 196 |  | 303 | 137 |  |
|  | **Medium** | 156 | 86 | 70 |  | 97 | 60 |  |
|  | **High** | 43 | 22 | 21 | 0.891 | 28 | 16 | 0.244 |

**
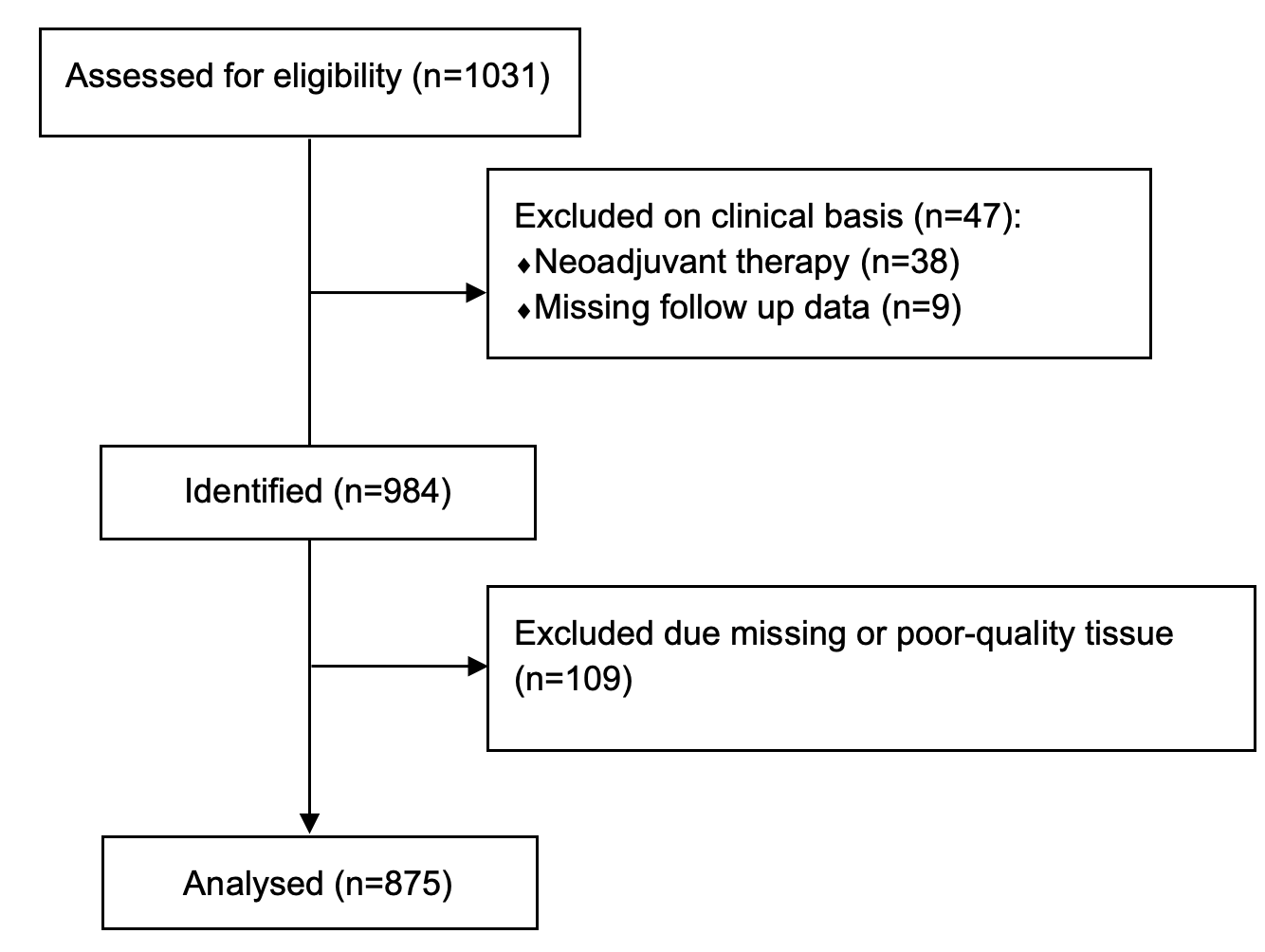
**

**Supplementary Figure 1. Study design.**

**
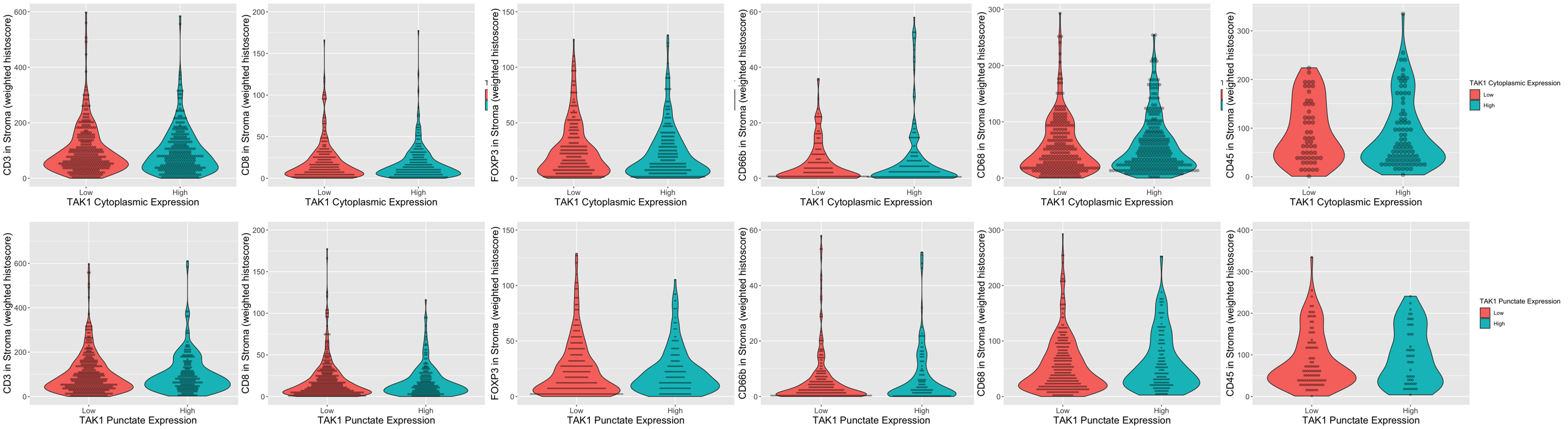
**

**Supplementary Figure 2.** Differential leukocyte composition within the tumour microenvironment based on TAK1 expression. High and low cytoplasmic TAK1 expression is demonstrated above, and high and low TAK1 punctate expression is shown below.

**Supplementary Figure 3.** Subgroup analysis comparing cancer-specific survival between patients with high and low cytoplasmic (top) and punctate TAK1 expression for neoadjuvant therapy subgroups (left) and adjuvant therapy subgroups (right). P-value calculated using log-rank test.

**Supplementary Figure 4.** Influence of TAK1 cytoplasmic and punctate expression on survival after colorectal cancer resection stratified by tumour location and microsatellite-stability status.

*Note: there were insufficient patient numbers I cohorts for MSI-high rectal cancers thus plots have not been illustrated.*

**
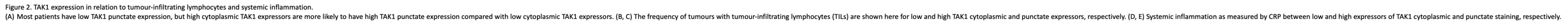
Supplementary Figure 5.** TAK1 expression in relation to tumour-infiltrating lymphocytes and systemic inflammation.

(A) Most patients have low TAK1 punctate expression, but high cytoplasmic TAK1 expressors are more likely to have high TAK1 punctate expression compared with low cytoplasmic TAK1 expressors. (B, C) The frequency of tumours with tumour-infiltrating lymphocytes (TILs) are shown here for low and high TAK1 cytoplasmic and punctate expressors, respectively. (D, E) Systemic inflammation as measured by CRP between low and high expressors of TAK1 cytoplasmic and punctate staining, respectively.

**Supplementary Figure 6.** Comparison of transcriptional differences between high and low cytoplasmic TAK1 expression. (A) Volcano plot of differential gene expression. (B) GSEA plots comparing low and high cytoplasmic TAK1 expression. (C) Heatmap of gene set enrichments of differential Hallmark patterns. (D) Deconvoluted microenvironment cell populations between conditions.

**Supplementary Figure 7.** Bulk RNA sequencing analysis based on punctate TAK1 immunostaining highlighting differential expression of IGF2 (A), PCA plots comparing low and high punctate TAK1 expression (B), Volcano plot using adjusted p-values (C), and significantly different hallmarks of cancer between low and high punctate TAK1 expression (D).

**Supplementary Figure 8.** Digital Assessment of Cytoplasmic TAK1 expression in colorectal cancer. Representative classifier for tumour and stroma by using QuPath digital pathological analysis (Left). Histogram of Cytoplasmic TAK1 expression as determined by QuPath digital pathological analysis. High and low expressors were determined based on mean histoscore (63.08) (Right).

**Supplementary Material (B):** GPOL 151-gene panel for mutational analysis

| ABCB1 | CSF1R | FGFR4 |  |
| --- | --- | --- | --- |
| KMT2A | PHF6 | SLC22A1 |  |
| ABCC2 | CTNNB1 | FLT1 |  |
| KRAS | PIK3CA | SLC22A2 |  |
| ABL1 | CYP19A1 | FLT3 |  |
| LAMA2 | PIK3R1 | SLC31A1 |  |
| ABL2 | CYP2A6 | FLT4 |  |
| LCK | PSMB1 | SLC34A2 |  |
| AKT1 | CYP2B6 | FSTL5 |  |
| LTK | PSMB2 | SLC45A3 |  |
| AKT2 | CYP2C19 | GNA11 |  |
| MAP2K1 | PSMB5 | SLCO1B1 |  |
| AKT3 | CYP2C9 | GNAQ |  |
| MAP2K2 | PSMD1 | SMAD4 |  |
| ALK | CYP2D6 | GNAS |  |
| MAP2K4 | PSMD2 | SMARCA4 |  |
| APC | DDR1 | GSTP1 |  |
| MAP3K1 | PTCH1 | SMARCB1 |  |
| ASXL1 | DDR2 | H3F3A |  |
| MAPK1 | PTEN | SMO |  |
| ATM | DDX3X | HNF1A |  |
| MED13 | PTPN11 | SNCAIP |  |
| ATRX | DNMT3A | HRAS |  |
| MET | RAF1 | SOS1 |  |
| BRAF | DPYD | IDH1 |  |
| MLH1 | RARA | SPRED1 |  |
| BRCA1 | EGFR | IDH2 |  |
| MPL | RARB | SRC |  |
| BRCA2 | ERBB2 | IKZF1 |  |
| MST1R | RARG | STK11 |  |
| CBL | ERBB3 | IL2RA |  |
| MTOR | RB1 | SUFU |  |
| CDA | ERBB4 | IL2RB |  |
| MYC | RET | TAS2R38 |  |
| CDH1 | ERG | IL2RG |  |
| MYD88 | ROS1 | TET2 |  |
| CDKN2A | ESR1 | INPP4B |  |
| NELL2 | RPS6KB1 | TP53 |  |
| CDKN2B | ESR2 | JAK1 |  |
| NF1 | RUNX1 | TRRAP |  |
| CEBPA | EZH2 | JAK2 |  |
| NOTCH1 | RXRA | TYK2 |  |
| CHD7 | FBXW7 | JAK3 |  |
| NPM1 | RXRB | UGT1A1 |  |
| CHIC2 | FGFR1 | KDM6A |  |
| NRAS | RXRG | VHL |  |
| FGFR2 | KDR | PDGFRA |  |
| SHH | WT1 | CRLF2 |  |
| FGFR3 | KIT | PDGFRB |  |
| SHOC2 | YES1 | ZMYM3 | CREBBP |
