# Supplementary material for: TAK1 expression is associated with increased PD-L1 and decreased cancer-specific survival in microsatellite-stable colorectal cancer
Source: Transl Oncol. 2024 Jul 27;48:102064. doi: 10.1016/j.tranon.2024.102064 (PMC11338118; doi:10.1016/j.tranon.2024.102064)
Supplement: Supplementary file 1 [file mmc1.pdf]

Decision on AJPA-D-24-00184

em.ajpa.0.89bb4f.4d2ec313@editorialmanager.com
<em.ajpa.0.89bb4f.4d2ec313@editorialmanager.com>
on behalf of
The American Journal of Pathology <em@editorialmanager.com>
Thu 29/02/2024 22:13

To:Norman Galbraith <Norman.Galbraith@glasgow.ac.uk>

CC: "Jean A. Quinn" jean.quinn@glasgow.ac.uk, "Sara Sf Al-Badran" s.al-badran.1@research.gla.ac.uk, "Kathryn A.F. Pennel" kathryn.pennel@glasgow.ac.uk, "Lily V.S. Hillson" 2716373h@student.gla.ac.uk, "Phimmada Hatthakarnkul" phimmada.hatthakarnkul@glasgow.ac.uk, "Molly McKenzie" m.mckenzie.1@research.gla.ac.uk, "Noori Maka" noori.maka@nhs.scot, "Lynette Loi" lynetteloi_77@hotmail.com, "Mikaela Frixou" mikaela.frixou@gmail.com, "Colin W. Steele" colin.steele@glasgow.ac.uk, "Campbell S. Roxburgh" campbell.roxburgh@glasgow.ac.uk, "Paul G. Horgan" paul.horgan@glasgow.ac.uk, "Donald C. McMillan" donald.mcmillan@glasgow.ac.uk, "Joanne Edwards" joanne.edwards@glasgow.ac.uk

AJPA-D-24-00184

Punctate TAK1 expression is associated with decreased cancer-specific survival in colorectal cancer

Dear Dr Galbraith,

Thank you for submitting your manuscript to The American Journal of Pathology for consideration.

The manuscript underwent review by the Editors, who regrettably were unable to consider it further. Although the Editors recognize that your study contains valuable information, they agreed that its overall descriptive nature and focus on prognosis do not align well with The American Journal of Pathology’s emphasis on pathogenesis and would not achieve a priority score necessary for publication. Priority is determined by Editors' recommendations and an assessment of the manuscript relative to other papers being considered. The publication priority assigned to each manuscript is, therefore, a critical factor in the evaluation of manuscripts.

Given the large number of manuscripts submitted to the journal, we must be selective in which are chosen for full review. Please be aware that although your manuscript was not sent to external reviewers for further consideration, it was thoroughly evaluated by the Editors. We hope that by providing you with an expedited decision, it may accelerate your ability to submit to another journal.

We are sorry that we were unable to accommodate this manuscript, but your interest in The American Journal of Pathology is appreciated. We hope that you will consider the Journal for future submissions.

Sincerely,

Martha B. Furie, PhD
Editor in Chief
The American Journal of Pathology

A copy of this decision has been sent to all co-authors.

For alternative journals that may be more suitable for your manuscript, please refer to our Journal Finder (https://eur03.safelinks.protection.outlook.com/?url=http%3A%2F%2Fjournalfinder.elsevier.com%2F&data=05%7C02%7CNorman.Galbraith%40glasgow.ac.uk%7Cf2f47764a0964cca754508dc3973b053%7C6e725c29763a4f5081f22e254f0133c8%7C1%7C0%7C638448416233653344%7CUnknown%7CTWFpbGZsb3d8eyJWIjoiMC4wLjAwMDAiLCJQIjoiV2luMzIiLCJBTiI6Ik1haWwiLCJXVCI6Mn0%3D%7C0%7C%7C%7C&sdata=%2F2Dgf7a8q51R%2F3cXCL3Q2LbYpPxx%2BxgxzjrKx6SBkd8%3D&reserved=0).

Have questions or need assistance?

For questions relating to your manuscript, the review process, or production, please contact the editorial office directly at ajp@asip.org.

For questions related to the Editorial Manager interface, please visit our customer service site: https://eur03.safelinks.protection.outlook.com/?url=http%3A%2F%2Fhelp.elsevier.com%2Fapp%2Fanswers%2Flist%2Fp%2F9435%2F&data=05%7C02%7CNorman.Galbraith%40glasgow.ac.uk%7Cf2f47764a0964cca754508dc3973b053%7C6e725c29763a4f5081f22e254f0133c8%7C1%7C0%7C638448416233660601%7CUnknown%7CTWFpbGZsb3d8eyJWIjoiMC4wLjAwMDAiLCJQIjoiV2luMzIiLCJBTiI6Ik1haWwiLCJXVCI6Mn0%3D%7C0%7C%7C%7C&sdata=zJH9tsC9qo4rGAY6LeOPDaYVEws2f%2BeaOD3d3a5RV0M%3D&reserved=0. Here you can search for solutions on a range of topics, find answers to frequently asked questions, and learn more about Editorial Manager via interactive tutorials. You can also talk 24/5 to our customer support team by phone and 24/7 by live chat and email.

At Elsevier, we want to help all our authors to stay safe when publishing. Please be aware of fraudulent messages requesting money in return for the publication of your paper. If you are publishing open access with Elsevier, bear in mind that we will never request payment before the paper has been accepted. We have prepared some guidelines (https://eur03.safelinks.protection.outlook.com/?url=https%3A%2F%2Fwww.elsevier.com%2Fconnect%2Fauthors-update%2Fseven-top-tips-on-stopping-apc-scams&data=05%7C02%7CNorman.Galbraith%40glasgow.ac.uk%7Cf2f47764a0964cca754508dc3973b053%7C6e725c29763a4f5081f22e254f0133c8%7C1%7C0%7C638448416233663733%7CUnknown%7CTWFpbGZsb3d8eyJWIjoiMC4wLjAwMDAiLCJQIjoiV2luMzIiLCJBTiI6Ik1haWwiLCJXVCI6Mn0%3D%7C0%7C%7C%7C&sdata=%2FBmAGCwvGvvvchyp%2FwSNQB6%2Fgkd8kIBYFk%2FznOOhbt4%3D&reserved=0) that you may find helpful, including a short video on Identifying fake acceptance letters (https://eur03.safelinks.protection.outlook.com/?

url=https%3A%2F%2Fwww.youtube.com%2Fwatch%3Fv%3Do5l8thD9XtE&data=05%7C02%7CNorman.Galbraith%40glasgow.ac.uk%7Cf2f47764a0964cca754508dc3973b053%7C6e725c29763a4f5081f22e254f0133c8%7C1%7C0%7C638448416233666888%7CUnknown%7CTWFpbGZsb3d8eyJWIjoiMC4wLjAwMDAiLCJQIjoiV2luMzIiLCJBTiI6Ik1haWwiLCJXVCI6Mn0%3D%7C0%7C%7C%7C&sdata=NZGX1CodJfW9lyHwvp3qOxSKmRK8%2FP6NSAq3Sve8xyg%3D&reserved=0). Please remember that you can contact Elsevier?s Researcher Support team (https://eur03.safelinks.protection.outlook.com/?url=https%3A%2F%2Fservice.elsevier.com%2Fapp%2Fhome%2Fsupporthub%2Fpublishing%2F&data=05%7C02%7CNorman.Galbraith%40glasgow.ac.uk%7Cf2f47764a0964cca754508dc3973b053%7C6e725c29763a4f5081f22e254f0133c8%7C1%7C0%7C638448416233669837%7CUnknown%7CTWFpbGZsb3d8eyJWIjoiMC4wLjAwMDAiLCJQIjoiV2luMzIiLCJBTiI6Ik1haWwiLCJXVCI6Mn0%3D%7C0%7C%7C%7C&sdata=pr%2FrtXPDSKhLfXw44SJQFiqkqPlnWtkLKNlD7Hb3jL4%3D&reserved=0) at any time if you have questions about your manuscript, and you can log into Editorial Manager to check the status of your manuscript (https://eur03.safelinks.protection.outlook.com/?url=https%3A%2F%2Fservice.elsevier.com%2Fapp%2Fanswers%2Fdetail%2Fa_id%2F29155%2Fc%2F10530%2Fsupporthub%2Fpublishing%2Fkw%2Fstatus%2F&data=05%7C02%7CNorman.Galbraith%40glasgow.ac.uk%7Cf2f47764a0964cca754508dc3973b053%7C6e725c29763a4f5081f22e254f0133c8%7C1%7C0%7C638448416233672830%7CUnknown%7CTWFpbGZsb3d8eyJWIjoiMC4wLjAwMDAiLCJQIjoiV2luMzIiLCJBTiI6Ik1haWwiLCJXVCI6Mn0%3D%7C0%7C%7C%7C&sdata=hMQmIzf1t7MNY5KfjV457%2FrzFOjjjpe10h80AK71yV4%3D&reserved=0).

In compliance with data protection regulations, you may request that we remove your personal registration details at any time. (Use the following URL: https://eur03.safelinks.protection.outlook.com/?url=https%3A%2F%2Fwww.editorialmanager.com%2Fajpa%2Flogin.asp%3Fa%3Dr&data=05%7C02%7CNorman.Galbraith%40glasgow.ac.uk%7Cf2f47764a0964cca754508dc3973b053%7C6e725c29763a4f5081f22e254f0133c8%7C1%7C0%7C638448416233675771%7CUnknown%7CTWFpbGZsb3d8eyJWIjoiMC4wLjAwMDAiLCJQIjoiV2luMzIiLCJBTiI6Ik1haWwiLCJXVCI6Mn0%3D%7C0%7C%7C%7C&sdata=S0q8cM7KpreaRAcVh1bQu7KZfW3yhdKjNLwSGV8HI4E%3D&reserved=0). Please contact the publication office if you have any questions.
